# Universal Ecological Patterns in College Basketball Communities

**DOI:** 10.1371/journal.pone.0017342

**Published:** 2011-03-09

**Authors:** Robert J. Warren, David K. Skelly, Oswald J. Schmitz, Mark A. Bradford

**Affiliations:** School of Forestry and Environmental Studies, Yale University, New Haven, Connecticut, United States of America; University of Oxford, United Kingdom

## Abstract

The rank abundance of common and rare species within ecological communities is remarkably consistent from the tropics to the tundra. This invariant patterning provides one of ecology's most enduring and unified tenets: most species rare and a few very common. Increasingly, attention is focused upon elucidating biological mechanisms that explain these species abundance distributions (SADs), but these evaluations remain controversial. We show that college basketball wins generate SADs just like those observed in ecological communities. Whereas college basketball wins are structured by competitive interactions, the result produces a SAD pattern indistinguishable from random wins. We also show that species abundance data for tropical trees exhibits a significant-digit pattern consistent with data derived from complex structuring forces. These results cast doubt upon the ability of SAD analysis to resolve ecological mechanism, and their patterning may reflect statistical artifact as much as biological processes.

## Introduction

**Figure 1:**
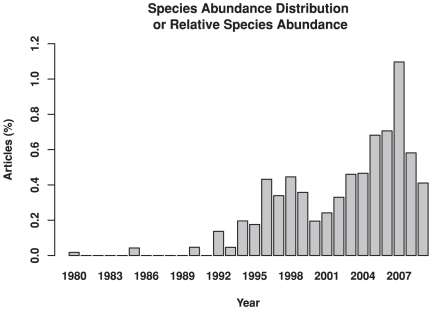
ISI's Web of Science reports a considerable increase in scientific articles containing the search terms “species abundance distribution” or “relative species abundance.” This increase represents the burgeoning debate about how ecological communities are structured. Shown is the percentage of articles with the search terms relative to all articles in the Biology and Ecology subject areas.

The species composition of ecological communities is as varied as the biophysical conditions where they occur. Accordingly, there is a prevailing sentiment that a general understanding of mechanisms leading to patterns in communities will be difficult if not impossible because communities are riddled by complexity, context-dependency and idiosyncrasy. It is therefore quite remarkable that a comparatively simple species abundance distribution (SAD) model ably describes pattern in widely divergent communities, making it one of ecology's most enduring tenets. Put simply, the rank abundance of constituent species is dominated by many rare and a few highly abundant species, regardless of community type [1], [2], [3]. Given the generality of the pattern, dozens of statistical models have been fit to SAD data to identify the elusive “silver-bullet” mechanism(s) driving the pattern [2], [4], [5], [6], [7], [8], [9]. Intense debate continues regarding which models fit best, how goodness-of-fit is measured and how to interpret successful or failed fits [2]. Indeed, the use of SAD patterns to explain ecological patterns and evaluate theory is increasing (Fig. 1). The most recent incarnation of the debate centers on whether or not the unified neutral theory (UNT) [1] sufficiently explains the pattern without the need to invoke non-neutral mechanisms.

We argue here that the ecological processes that structure natural communities cannot be determined by fitting models to SADs alone because they are generated from observational data, for which the underlying mechanisms are unknown [10], [11]; That is, there is not a benchmark to know what pattern the mechanism should produce. SAD patterns may instead merely reflect the vagaries of sampling and statistical properties of data [2], [9], [12], [13] that may lead to spurious conclusions about underlying mechanisms. Indeed, researchers have shown that the methods used to test hypotheses and generate SAD patterns can produce similar patterns from non-ecological, apparently random, data [12], [14]. These findings suggest that a universal mechanism structures ecological and non-ecological patterns, with the underlying mechanism unknown in both cases, or that the pattern is unrelated to underlying mechanisms.

## Methods

Whereas previous researchers have generated SAD patterns from non-ecological data to illustrate SAD shortcomings, these have incorporated processes with mechanisms as cryptic as those in ecological systems (e.g., SAD patterns in stock prices) [12]. Instead, we ask whether the SAD pattern can be generated from a data set structured by a known mechanism – not to infer the mechanics underlying ecological processes, but to examine the potential for SAD analysis to elucidate them. We do this by using the distribution of wins in college basketball games where the mechanism, “competitive exclusion,” is understood [15]. Competition in college basketball evolves from selection at all levels of organization. Universities invest heavily in salaries and facilities to attract top coaches and players, coaches invest long hours into rigorous recruiting and training the best players, and players invest many years toward improving skills and athleticism [16]. The end result is a community of teams with competitive edge skewed toward a few dominant teams that consistently win in head-to-head competition [15], [17]. Historically strong teams remain strong, whereas – with some variance – smaller schools in smaller conferences remain weak. Head-to-head competition structures the win-loss records of these teams, which play more often at regional scales with less common long-distance games. This competition creates a win-loss data set for which we know more about the structuring mechanism than for ecological community data, or for previous non-ecological data sets used to criticize SADs.

**Figure 2:**
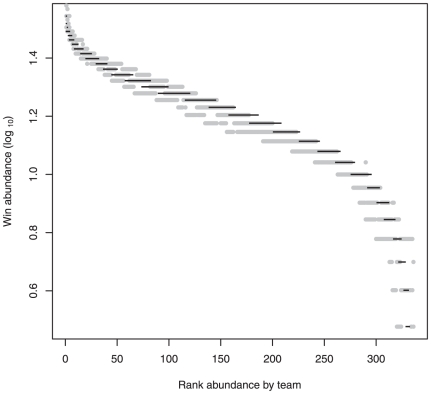
Rank abundance of college basketball wins by team. The abundance of wins in college basketball, a result of competition between teams of unequal abilities, creates the same pattern used by ecologists to infer mechanism from species abundance distributions (SADs). The log_10_ abundance of college basketball wins is ranked by team, just as the abundance of individuals is ranked by species for ecological communities. Mean wins (gray) across 2004 to 2008±95% CI are given along with random (*Normal*, µ = 16, σ = 6) wins (black), and these random and observed patterns are not significantly different (see text).

For ecological analogy, we treat each team as a species and each win as an individual of that species occupying a site. We explore the patterning that emerges and relate it to classic SADs. College basketball provides little insight into ecological processes, but it does provide an intuitive framework to examine the universality of the SAD pattern and its connection with a known structuring mechanism. We analyze win-loss records for 327 NCAA Division I teams (years 2004–2008 for statistical replication). We consider each team a species, and each win an individual (total wins equals species abundance). College basketball data are consistent with assumptions outlined for the UNT [1]. They follow a zero-sum gain as a win (*n*+1) by one team results in loss (*n*–1) by another (i.e. gain of an individual by one species results in loss of another species individual; and in college basketball teams cannot ‘draw’ a game). Further, there is a high species (team) richness and a high number of individuals (∼5,000 wins yr^−1^) competing on a single ‘trophic’ level. We rank abundance of wins per team (2004–2008, mean ±95% CI) creating a relative abundance distribution (Fig. 2). This is the classic method for empirically representing commonness and rarity in communities. We fit both the empirical and random data sets to a sigmoid curve using the nls() package [18] in the R statistical program [R Development Core Team 19].

## Results and Discussion

The rank abundance distribution shows a classic left-skewed pattern interpretable as a community characterized by few abundant species and many rarer species (Fig. 2). We generate the same pattern (goodness-of-fit, *r* ^2^ = 0.99) using five random season of college basketball wins (Fig. 2). The estimated asymptote (asym), inflection point (xmid) and curve steepness (scal) for the empirical and random data were a significant (p<0.001) fit to a sigmoid curve (asym/1+exp(-(xmid-data)/scal)). More importantly, the estimated parameters (±SE) for the empirical (asym: 27.3±0.3; xmid: 5.2±0.02; and scal: −0.7±0.02) and random (asym: 26.2±0.3; xmid: 5.3±0.02; and scal: −0.6±0.02) data did not differ significantly (Fig. 2).

These results demonstrate that a non-ecological dataset (college basketball) with a known mechanism (competition), and where there is also some ‘stochasticity’ (i.e. the favorite does not always win), generates a pattern purported to arise for communities from underlying ecological processes [9], [12], [20]. Moreover, randomly generated data produce the same pattern (Fig. 2). We can draw two important conclusions from these results. First, fitting niche or neutral models to this pattern – or to deviations from this pattern in a null framework – cannot deduce mechanism because even data with a known mechanism does not produce a SAD pattern that deviates from random. SAD patterns may be a universal product of large data sets and sampling artifacts, and this means they cannot truly be falsified – making this approach uncertain for hypothesis testing and model fitting. Second, we know that mechanism matters in college basketball as the powerhouse teams from the top conferences typically dominate, and the top teams are predictable based on their “traits”. In college basketball, such traits include the athletic department budget, the facilities, the coach's salary and the ability to attract top recruits [15], [21]. These traits are unequally distributed toward a few dominant teams, and these teams achieve a disproportionate number of wins. Yet, the outcome is a SAD pattern indistinguishable from random wins and most ecological communities.

These results beg the question: what processes underlie patterns of species distributions where more biological complexity occurs? Neutral [1] and niche [10] based approaches are considered alternative theories in community ecology (but see [22] for reconciliation), particularly because the universal nature of SAD patterning suggests that invoking niche differentiation is unnecessary in explaining community structuring [1]. The frequency distribution of species reflects numerical abundance, but assumes all species utilize resources similarly, share the same body size and interact equally [23], an assumption consistent with unified neutral theory [24], but commonly violated in natural communities [25]. Instead, it appears that the rule of large numbers, as noted by May [13], generates the SAD patterns for ecological communities and college basketball records. As we noted, college basketball teams do not share and utilize resources equally, and there is no reason to assume members of ecological communities do. Likewise, SAD patterns have been used as evidence of niche partitioning [8], [26], but if they are generated by combinations of complex factors in large data sets – or even many small random effects [27] – rather than underlying biological mechanisms, they also provide no falsifiable evidence of niche apportionment. For example, the significant-digit (aka Benford's) law stipulates that the first digit of non-random data sets with numbers that span several orders of magnitude are biased toward lower values [28], [29]. As a result, data sets ranging from sports statistics to river size usually contain numbers that predominately begin with 1 (30%), followed by numbers that begin with 2 (18%), 3 (13%) down to numbers beginning with 9 (5%) [proportion digit_x_ = log_10_ (digit_x+1_/digit_x_)] [29], [30]. This prompted us to investigate the concordance between an empirical species abundance data set, 319 tropical trees >10 cm DBH at Barro Colorado Island [31], and Benford's law. A chi-square test examining the difference between observed species abundance digit distribution and the expected Benford distribution shows that the BCI data follow the significant-digit pattern (*χ^2^* = 3.22, *df* = 8, *p* = 0.920). Whereas Newcomb and Benford based their findings on empirical observations [28], [29], Hill [30] offers a theoretical basis for the pattern. Essentially, the greater the complexity of interacting processes underlying a data set, the more the first digits converge to a logarithmic distribution as described by Benford's law [30]. That BCI tree species abundance follows Benford's law indicates that the pattern may reflect multiple and complex ecological mechanisms, and this possibility further undermines SAD usefulness as substantiation for overarching theories.

Ecological stalwarts such as MacArthur [32] and May [13] long ago questioned the use of SAD patterns in ecological analysis, even going so far to call it an “obsolete approach to community ecology” [32]. Substantial evidence suggests the SAD pattern represents statistical and sampling artifacts equally as well as any structuring mechanism [12], [14], [20], making their ecological validity difficult to assess (Fig. 2). Whilst we do not provide nor posit a proof that SAD fitting fails to adequately represent ecological communities, we provide sufficient evidence that SAD patterns may (1) derivate from purely statistical or sampling processes and/or (2) oversimplify and obfuscate complex ecological dynamics. The escalation in the use and analysis of SAD patterns may represent a substitution of statistical elegance for ecological relevance. After almost 80 years of attempts to explain SADs, with equivocal results [2], [9], this pattern fitting persists within the ecological milieu and even has increased in recent decades (Fig. 1), e.g., [33]. Our findings do not resolve niche vs. neutral debates, nor do we shed light on the mechanisms underlying ecological processes that structure communities, but we do highlight the critical need for field research rather than SAD patterning to test competing hypotheses explaining community patterns [9], [10], [34]. This may require acceptance that ecological systems are cryptic and complex and not easily synthesized to fit simple overarching models [10], [34]. This approach requires improved integration of empirical and theoretical ecology with direct experimental evidence of putative structuring mechanisms to evaluate the niche and/or neutral processes structuring ecological communities.
